# PP2A Deficiency Enhances Carcinogenesis of Lgr5^+^ Intestinal Stem Cells Both in Organoids and In Vivo

**DOI:** 10.3390/cells9010090

**Published:** 2019-12-30

**Authors:** Yu-Ting Yen, May Chien, Yung-Chih Lai, Dao-Peng Chen, Cheng-Ming Chuong, Mien-Chie Hung, Shih-Chieh Hung

**Affiliations:** 1 Drug Development Center, Institute of New Drug Development, China Medical University, Taichung 40402, Taiwan; d92449001@ntu.edu.tw (Y.-T.Y.); maymaychien2k12@gmail.com (M.C.); 2 Integrative Stem Cell Center, China Medical University Hospital, Taichung 40402, Taiwan; yungchihlai@gmail.com (Y.-C.L.); cmchuong@med.usc.edu (C.-M.C.); 3 Kim Forest Enterprise Co., Ltd., Taipei 22175, Taiwan; D96743@mail.cmuh.org.tw; 4 Department of Pathology, Keck School of Medicine, University of Southern California, Los Angeles, CA 90033, USA; 5 Center for Molecular Medicine and Graduate Institute of Cancer Biology, China Medical University, Taichung 40402, Taiwan; mhung77030@gmail.com; 6 Cancer Biology Program, The University of Texas Graduate School of Biomedical Sciences, The University of Texas MD Anderson Cancer Center, Houston, TX 77030, USA; 7 Department of Orthopaedics, China Medical University Hospital, Taichung 40402, Taiwan

**Keywords:** carcinogen, protein phosphatase 2A (PP2A), intestinal tumor, intestinal organoid, Lgr5^+^ crypt stem cell

## Abstract

In most cancers, cellular origin and the contribution of intrinsic and extrinsic factors toward transformation remain elusive. Cell specific carcinogenesis models are currently unavailable. To investigate cellular origin in carcinogenesis, we developed a tumorigenesis model based on a combination of carcinogenesis and genetically engineered mouse models. We show in organoids that treatment of any of three carcinogens, DMBA, MNU, or PhIP, with protein phosphatase 2A (PP2A) knockout induced tumorigenesis in Lgr5^+^ intestinal lineage, but not in differentiated cells. These transformed cells increased in stem cell signature, were upregulated in EMT markers, and acquired tumorigenecity. A mechanistic approach demonstrated that tumorigenesis was dependent on Wnt, PI3K, and RAS-MAPK activation. In vivo combination with carcinogen and PP2A depletion also led to tumor formation. Using whole-exome sequencing, we demonstrate that these intestinal tumors display mutation landscape and core driver pathways resembling human intestinal tumor in The Cancer Genome Atlas (TCGA). These data provide a basis for understanding the interplay between extrinsic carcinogen and intrinsic genetic modification and suggest that PP2A functions as a tumor suppressor in intestine carcinogenesis.

## 1. Introduction

The cells of origin in most cancers have remained unknown. Chemical carcinogenesis mouse models recapitulating most of human cancers that are induced by exposure to environmental carcinogens [1], however, is difficult to be achieved in a cell-specific manner. Therefore, the current strategies to investigate the cellular origins of cancers are using genetically engineered mouse models (GEMMs), with either transgenic or conditionally targeted gene technologies to induce tumor in different cellular contexts [2]. Moreover, both models take a long time to develop cancer, limiting progress in the cancer research field.

The most applied animal model for studying intestinal tumorigenesis is based on activating mutations in the Wnt pathway, which relies on adenomatous polyposis coli (Apc) depletion [3] and beta-catenin (CTNNB1) activation [4], leading to beta-catenin stabilization and constitutive transcription of its down-stream genes. Recent progress in the understanding of the cell of origin of intestinal tumor was made using this model, although several inconsistencies were observed. After in vivo Apc depletion in leucine-rich-repeat containing G-protein-coupled receptor 5 (Lgr5)^+^ crypt stem cells, tumor formation occurred within 3–5 weeks [3]. However, Apc depletion or being combined with Kras^G12D^ mutation in progenitor and differentiated cells did not induce tumor formation [5,6]. However, tumor-initiating mutations can occur in Lgr5^+^ crypt stem cells and in differentiated Lgr5^−^ cells [4], indicating that the two hypotheses are not mutually exclusive. Ablation of Lgr5^+^ cells in orthotopically transplanted tumors, generated by genetic modification in differentiated villus cells, suppressed tumor growth [7]. Interestingly, Lgr5^+^ cells reappeared and tumors recurred when ablation was terminated 4 days later. The generation of Lgr5^+^ cells from Lgr5^−^ cells after Lgr5^+^ ablation was also observed in the xenograft mouse model of human colon cancer stem cells (CSCs) [8]. However, the specific mechanism of Lgr5^+^ cell generation from remaining Lgr5^−^ cells remains unclear.

Aberrant activation of signal transduction pathway, a dynamic process involving an ‘on/off’ switch, can transform a normal cell to be malignant or further render cancer cells with the capacities for therapy resistance. Activating mutations in genes encoding kinases or signaling molecules, such as RAS and PI3K, switch on the signaling, continuously activating a survival and/or proliferation pathway, while activations of phosphatases, such as the serine/threonine phosphatase PP2A family, switch off the signaling [9]. Previous efforts through high-throughput screens of tyrosine kinome and tyrosine phosphatome have identified several driver or passenger mutations in a spectrum of malignancies, including intestinal tumor [10,11,12]. However, there are few if any studies focusing on the altered signalings driven by serine/threonine kinase mutations [13]. Human intestinal tumors contain active mutations in genes encoding proteins involved in the WNT, MAPK, TGF-beta, and PI3K pathways [14]. Ingenuity pathway analysis (IPA) of TCGA-COAD revealed PP2A complex and its subunits, such as PPP2R1A and PPP2CA, are intercalated among several driver mutation pathways (Figure S1A–C). Moreover, endogenous PP2A inhibitors, SET and CIP2A [15], are highly expressed in intestinal tumors in comparison to their matched normal tissue samples (Figure S1D). We showed that PP2A was suppressed in intestinal tumor stem cells (CSCs), thereby activating its substrate kinases to enhance survival under hypoxia and serum depletion [16], thereby increasing resistance to anti-angiogenesis therapy [17]. Our recent studies also demonstrated that reduced PP2A activity in colorectal and lung CSCs enhances suspension survival and induces tumor initiation [18], revealing the tumor suppressive role of PP2A [19]. Although higher numbers of Apc, p53, Kras^G12D^, and Smad4 driver mutations may be required for human colorectal tumorigenesis, there are some intestinal tumors carry only one or no alteration in these driver mutations [20]. For example, gene fusions involving R-spondin 1 occurring in 10% of intestinal tumor are mutually exclusive with active Wnt signaling caused by *APC* or *CTNNB1* mutations [20]. The emerging novel intestinal tumorigenesis animal models should allow for elucidating the molecular mechanisms of these cancers.

Given that cancer is the product of complex interactions between the genetic and environmental predisposition factors, the combined use of chemical carcinogens that switch on kinases and GEMM with phosphatase deficiency is a logical approach for examining the complex interplay between genetic susceptibility and environmental exposure [21]. To investigate the cell origin of intestinal tumor, we first combined treatment with carcinogen 7,12-dimethylbenzanthracene (DMBA) that has previously been known to induce rodent s in the presence of 1,2-dimethyl-hydrazine [22] and PP2A inhibition via okadaic acid (OA) treatment or genetic deficiency. DMBA not only activates multiple mutations in different codons of ras [23] but also induces activation in other pathways, such as Notch [24], providing a screening approach for identifying key kinases or molecules. Besides DMBA, we also investigated the effects of *N*-methyl-*N*-nitrosourea (MNU) and 2-amino-1-methyl-6-phenylimidazo-[4,5-b]pyridine (PhIP) on intestinal carcinogenesis. MNU is one of the direct alkylating agents, which does not require metabolic activation for initiating carcinogenesis [25]. PhIP has received considerable attention because it has multi-organ targets and it was developed upon broiling of fish and meat [26]. Moreover, we established primary intestinal organoid models that recapitulate the rodent intestinal tumorigenesis paradigm [27]. These rodent intestinal tumorigenesis models are useful in the development of new strategies for targeting rodent intestinal CSCs and treatment of intestinal tumor.

## 2. Materials and Methods

### 2.1. Mouse Colonies

*Ppp2r1a^flox/flox^* mice, carrying conditional alleles with loxP sites flanking exon 5–6 of *Ppp2r1a*, were purchased from the Jackson Laboratory and crossed to *Lgr5-EGFP-CreERT2* or *Villin-Cre* mice to generate *Lgr5-EGFP-CreERT2; Ppp2r1a^flox/flox^* or *Villin-Cre; Ppp2r1a ^flox/flox^* mice. NOD/SCID mice were purchased from Lasco Co., Ltd. (Taiwan). All animal studies and care of live animals were approved and performed following the guidelines made by the China Medical University Institutional Animal Care and Use Committee 2016-398-1; 2017-239.

### 2.2. Mouse Intestinal Organoid Cell Isolation, Culture, and Passage

Organoid culture was preformed according to a protocol modified from previously described methods [28]. In brief, the intestines were dissected, opened longitudinally and cut into small (2 mm) pieces. The tissues were rocked in dissociation reagent and incubated at room temperature (15–25 °C) for 15 min. The tissues were then mixed and filtered through a 70 μm sterile cell strainer. The crypts were collected by centrifugation at 140× *g* for 5 min at 4 °C. Approximately 500 crypts were suspended in 50 μL growth factor reduced phenol-free Matrigel (BD Biosciences, San Jose, CA, USA). Next, a 50 μL droplet of Matrigel/crypt mix was placed and polymerized in the center well of a 48-well plate. The basic culture medium (Dulbecco’s modified Eagle’s medium/F12 supplemented with penicillin/streptomycin), was supplemented with 50 ng/mL murine recombinant epidermal growth factor (EGF; Peprotech, Hamburg, Germany), Noggin (5% final volume) and R-spondin 1 (5% final volume) called ‘’ENR’’ medium. Medium change was performed every 3–4 days. Each condition was examined in triplicate with multiple (>15) organoids in each sample. Each experiment was repeated twice.

### 2.3. Dysplasia Index

Histologic changes were scored blindly on the levels of four histological characteristics as previously described [27]: nuclear grade (enlarged nuclei with diffuse membrane irregularities and prominent nucleoli); stratification; mitoses and invasion (>2 foci). The dysplasia index was evaluated by all microscopic fields containing viable organoids with 5 fields per sample (*n*).

### 2.4. Primary Organoid Transplantation

For transplantation, cells from passage 7; day 50 organoid cultures were collected. Dissociated cells were pelleted by centrifugation and resuspended with Matrigel (50% Matrigel (BD), in a total volume of 100 μL containing indicated cell numbers and injected s.c. into NOD-SCID mice.

### 2.5. Immunofluorescence and Immunohistochemistry

Freshly isolated intestines were prepared according to a protocol modified from previously described methods [27]. The intestines were then applied for immunostaining. For immunostaining, the organoid cells were rinsed three times in ice-cold PBS. The organoid cells were spun down at 900 rpm for 10 min at 4 °C. Sections were deparaffinized and stained with H&E for the initial histology analysis. The immunofluorescence was performed on paraffin-embedded sections (5 μm). The permeabilized organoid cell samples were incubated with primary antibodies overnight at 4 °C. The samples were incubated with anti-PPP2R1A (GTX102206; GeneTex, Hsinchu City, Taiwan), anti-CK20 (GTX110600 Genetex), anti-Lgr5 (GTX50839 Genetex), anti-SMA (Abcam, ab5694, Cambridge, MA, USA), anti-beta-catenin (BD Transduction Labs, San Jose, CA, USA; 610154); the secondary antibodies used were DyLight^®^ 650 Conjugated goat anti-rabbit (cat no. A120-101D5; Bethyl Laboratories Inc., Montgomery, TX, USA) and Goat anti-Rabbit IgG Antibody-FITC (Bethyl cat no. A120-101F) and DAPI (Molecular Probes) for 1 h at room temperature. The slides were mounted with SlowFade (SlowFade^®^ AntiFade Kit, Molecular Probes, Waltham, MA, USA) followed by covering with a coverslip, and the edges were sealed to prevent drying. The specimens were examined with a Zeiss 710 Laser Scanning confocal microscope (Zeiss, Oberkochen, Germany).

Intestinal tissue was fixed and processed into paraffin blocks according to standard procedures. beta-catenin immunohistochemistry was performed as previously described [3]. Immunohistochemistry protocol hold as following: freshly isolated intestines were flushed with 10% formalin in PBS and fixed by incubation in a 10-fold excess of formalin overnight at room temperature. The formalin was removed and the intestines washed twice in PBS at room temperature. The intestines were then transferred to a tissue cassette and dehydrated by serial immersion in 20-fold volumes of 70, 96 and 100% EtOH for 2 h each at 4 °C. Excess ethanol was removed by incubation in xylene for 1.5 h room temperature and the cassettes then immersed in liquid paraffin (56 °C) overnight. Paraffin blocks were prepared using standard methods and 4μm tissue sections generated. These sections were de-waxed by immersion in xylene (2 × 5 min) and hydrated by serial immersion in 100% EtOH (2 × 1 min), 96% EtOH (2 × 1 min), 70% EtOH (2 × 1 min) and distilled water (2 × 1 min). Endogenous peroxidase activity was blocked by immersing the slides in peroxidase blocking buffer (0.040 M citric acid, 0.121 M disodium hydrogen phosphate, 0.030 M sodium azide, 1.5% hydrogen peroxide) for 15 min at room temperature. For beta-catenin, antigen retrieval involved 20 min boiling in Tris-EDTA pH 9.0, and blocking buffer (1% BSA in PBS) added to the slides for 30 min at room temperature. For beta-catenin (BD Transduction Labs, 610154), staining involved 1/100 dilution in blocking buffer (0.05% BSA in PBS) for 2 h at room temperature. 

The slides were then rinsed in PBS and secondary antibody added (polymer HRP-labeled anti-mouse/rabbit, Envision) for 30 min at room temperature (Dako, Trappes, France). Slides were again washed in PBS and bound peroxidase detected by adding DAB substrate for 10 min at room temperature. Slides were then washed 2× in PBS and nuclei counterstained with Mayer’s hematoxylin for 2 min, followed by two rinses in distilled water. Sections were dehydrated by serial immersion for 1 min each in 50 and 60% EtOH, followed by 2 min each in 70, 96, and 100% EtOH and xylene. Slides were mounted in mounting medium and a coverslip placed over the tissue section.

For immunohistochemistry (IHC) analysis, nuclei expressing beta-catenin after IHC staining were counted under 200× magnification. The Histological score (H-score) was determined based on the intensity and percentage of nucleus staining at each intensity [29], and calculated as follows: H-score = (nucleus showing highly beta-catenin expression) × 3 + (nucleus showing beta-catenin expression) × 2 + (nucleus showing weak beta-catenin expression) × 1.

### 2.6. Viral Infection of Organoids

For in vitro deficiency of the *Ppp2r1a*, organoid cultures containing floxed *Ppp2r1a* alleles were infected with adenovirus-encoding Cre recombinase (Ad-Cre) (Vector Biolabs, Philadelphia, PA, USA) at a titer of 100 multiplicity of infection (MOI) [27]. 

### 2.7. Tamoxifen Induction

Mice aged 6–8 weeks were injected intraperitoneally with a single 200 μL dose of tamoxifen in sunflower oil at 10 mg/mL.

### 2.8. Organoid Disaggregation, FACS, and Immunoblotting

Organoid cultures were recovered and dissociated from collagen gel by collagenase IV incubation, followed by incubation with 0.05% trypsin and EDTA. After extensive washing with 10% FBS, cells were filtered with 40-μm cell strainers (BD Falcon) Pellets were resuspended with FACS staining solution (5% FCS in PBS). Stringent wash was applied using ice-cold PBS, followed by isolation of Lgr5^−^EGFP^+^ cells using an FACSAria II (BD) [30]. For immunoblotting, the organoid cells were lysed in lysis buffer (1% Triton X-100, 150 mmol/L NaCl, 10 mmol/L Tris pH 7.4, 1 mmol/L EDTA pH 8.0, protease inhibitor cocktail) and then sonicated. The protein concentration was then measured. Next, equal amounts of protein (20 μg/well) were separated by SDS-polyacrylamide gel electrophoresis, transferred to nitrocellulose, and immunoblotted with primary antibodies. The membranes were blocked with CISblock buffer purchased from Cis-biotechnology, Taiwan. The following antibodies were used: anti-phospho-AKT (Ser-473), anti-AKT; anti-phospho-ERK1/2 (Thr-202/Tyr-204), anti-ERK, and anti-PP2A from Cell Signaling; anti-Lgr5 and anti-alkaline phosphate intestinal (Alpi) (Genetex, epitope C-terminus), beta-catenin (BD Transduction Labs, 610154), anti-Villin (Santa Cruz, Dallas, TX, USA), and anti-beta-actin and GAPDH (Sigma-Aldrich, St. Louis, MO, USA). Following the primary antibody incubation, the nitrocellulose membranes were incubated with secondary antibodies and visualized by ECL.

### 2.9. Antibody Arrays of Mouse AKT Pathway Phosphorylation

The RayBio™ Mouse AKT Pathway Phosphorylation Array Kit (cat. no. AAH-AKT1-2) was purchased and preformed according to a protocol modified from RayBiotech Inc. (Norcross, GA, USA). Briefly, the array membranes were blocked with blocking buffer for 30 min at room temperature. The membranes were then incubated with 2 mL of lysate prepared from organoid cultures with different treatments after normalization with equal amounts of protein. After extensive washing with wash buffer I (3 washings of 5 min each), and wash buffer II (3 washings of 5 min each) to remove unbound materials, the membranes were incubated with the Detection Antibody Cocktail for 1.5 to 2 h at room temperature, followed by wash with wash buffer I and II. Then the membranes were incubated with HRP-Anti-Rabbit IgG for 2 h at room temperature. The unbound HRP antibody was washed out with wash buffer I and II. Finally, each array membrane was exposed to X-ray film using a chemiluminescence detection system (Perkin Elmer, Wellesley, MA, USA).

### 2.10. Transcriptome Analysis

RNA was extracted from organoid culture using an RNeasy Kit (Qiagen, Hilden, Germany). RNA integrity was assessed using the RNA Nano6000 assay kit (Agilent Technologies, Santa Clara, CA, USA). For RNA-seq, library preparation and sequencing were performed by Novogene Technology. The output data (FASTQ files) were mapped to the target genome (TopHat v2.0.12), which can generate a database of splice junctions based on the gene model annotation file. HTSeq v0.6.1 was used to count the reads numbers mapped to each gene. Then the FPKM of each gene was calculated based on the length of the gene and reads count mapped to this gene. Differential expression heatmap results and biological variability were analyzed by ClustVis free web server [31] and gene set enrichment analysis (GSEA) [32], respectively. Data were submitted and approved by Gene Expression Omnibus (GEO; accession number GSE120241).

### 2.11. Whole-Exome Sequencing, Alignment, and Annotation

Exome sequences were captured with SureSelect^XT^ Mouse All Exon Kit (G7550E-001, Agilent, CA, USA) following the standard protocols. The products of exome capture should pass criteria: the length of fragments: 300 ± 30 bp and total amount: >600 ng. After exome capturing, the index-tagged samples were pooled and sequenced on Illumina HiSeq 2000. Burrows-Wheeler Alignment (v0.7.12) was employed to align reads to the reference genome (mm10) with default parameters. Aligned reads were sorted by picard-tools (v1.8). The duplicated reads were marked by picard-tools. Indel Realignment were performed with GenomeAnalysisTK (v3.5) using mm10 dbsnp database as known sites. Base quality score recalibration was also performed with GenomeAnalysisTK (v3.5) using mm10 dbsnp database. SNPs and indels were called by GenomeAnalysisTK HaplotypeCaller (v3.5), which used default parameters. Whole exome sequencing raw data was submitted to SRA database (SRA; http://trace.ncbi.nlm.nih.gov/Traces/sra/, accession number SRP162613) 

### 2.12. Statistics

The *p*-values were determined using two-tailed Student’s *t*-test (t groups) and One-way ANOVA (>2 groups). A *p*-value less than 0.05 was considered significant.

## 3. Results

### 3.1. Combination of DMBA and OA Treatment Induces Dysplasia and Oncogenic Transformation in Organoid Culture 

**Figure 1:**
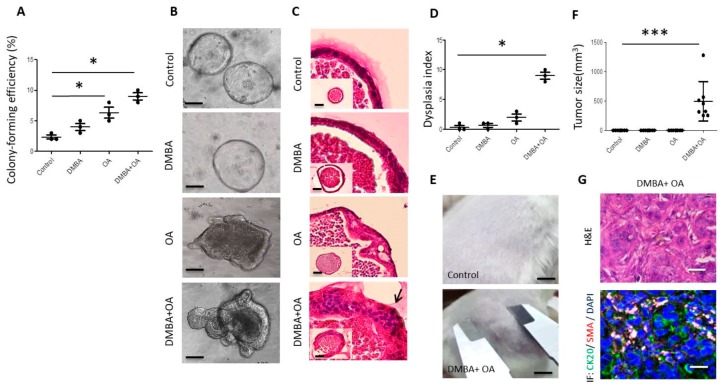
Combination of DMBA and okadaic acid (OA) induces dysplasia and oncogenic transformation in wild-type intestinal organoid culture. In vitro culture of wild type intestinal organoids without (control) or with DMBA or/and tamoxifen (TAM) in the presence of EGF, Noggin, and R-spondin 1 (500 single cells/well). (**A**) Colony (organoid)-forming efficiency was calculated at day 7. Experiment has been carried out in triplicate and each time 100 organoids were counted in each group. (**B**) Bright-field of organoid culture at day 50. Scale bar, 100 μm. (**C**) H&E staining and histologic characterization of cystic stratified epithelium with nuclear pleomorphism (arrow). Scale bar, 50 μm. (**D**) Dysplasia index at day 50 (experiment were repeated twice with *n* = 3 microscopic fields containing viable organoids). (**E**) Dissociated cells in Matrigel (500,000 cells/100 μL) were injected s.c. into NOD-SCID mice. In vivo tumor formation 45 days later (For those without tumor formation, observation was extended for up to 3 months, experiment were repeated twice with *n* = 3). (**F**) Tumor size 45 days after s.c. implantation (*n* = 8). (**G**) H&E staining and immunofluorescence of CK20 and SMA for tumor sections. Scale bar, 100 μm. *, *p* < 0.05; ***, *p* < 0.0001 as determined with one-way ANOVA.

We chose an organoid culture system supported by epidermal growth factor (EGF), Noggin and R-spondin 1 (ENR) medium to investigate whether DMBA or/and OA could induce oncogenic transformation. As previously described [27], small intestine or colon organoids predominantly exhibited a well-organized, stereotyped epithelial single-layer organization at 7 days of culture, and maintained the similar morphology over a 50-day period of culture (Figure 1). At day 7, DMBA did not affect colony (organoid)-forming efficiency. OA induced a slight increase in both colony-forming efficiency, while a combination of DMBA and OA induced a large and significant increase in colony-forming efficiency (Figure 1A). At day 50, DMBA did not affect organoid morphology, OA induced mild enlargement in part of the epithelial layer with crowded nuclei, while a combination of DMBA and OA induced a very large malformation involving the entire epithelium with a confluent sheet of nuclear pleomorphism (Figure 1B), similar to that observed only when combining *Apc*, *p53*, *Kras^G12D^*, and *Smad4* mutations in differentiated villus cells [27]. Histological analysis revealed that organoids treated with DMBA alone had a single-layer epithelium, similar to the control. OA-treated organoids showed multi-cell-layer-changes in only a small part of the epithelium, while those treated with DMBA in combination with OA showed multi-cell-layer-changes with loss of the cell border in nearly the entire epithelium (Figure 1C), similar to the histology achieved only by quadruple mutants, *Apc/Kras^G12D^/p53/Smad4* [27]. A dysplasia index quantification of proliferation, nuclear atypia, invasion, and cellular stratification in organoids indicated that DMBA did not induce dysplasia compared to the control, OA induced a marginal increase in dysplasia, while the combination of DMBA and OA induced a large and significant increase in dysplasia (Figure 1D). Furthermore, the combination of DMBA and OA, but not DMBA or OA alone, endowed organoids with robust in vivo tumorigenicity, forming alpha-smooth muscle actin (SMA)^+^ and CK20^+^ intestinal tumor after subcutaneous transplantation (Figure 1E–G).

### 3.2. Combination of DMBA Treatment and PP2A Deficiency Induces Dysplasia and Oncogenic Transformation in Organoid Culture

**Figure 2:**
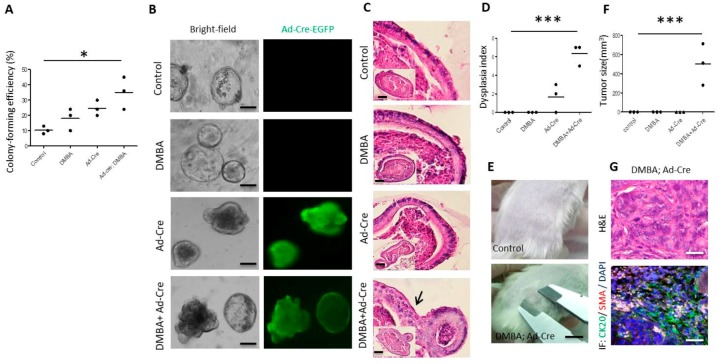
Combination of DMBA and Ad-Cre induces dysplasia and oncogenic transformation in Ppp2r1aflox/flox intestinal organoid culture. In vitro culture of Ppp2r1aflox/flox intestinal organoids without (control) or with DMBA or/and Ad-Cre-GFP (Ad-Cre) infection in the presence of EGF, Noggin and R-spondin 1 (500 single cells/well). (**A**) Colony (organoid)-forming efficiency was calculated at day 7. At least 100 organoids were counted in each group. (**B**) Bright-field and fluorescence images of organoid culture at day 50. Scale bar, 100 μm. (**C**) H&E staining and histologic characterization of cystic stratified epithelium with nuclear pleomorphism (arrow indicated). Scale bar, 50 μm. (**D**) Dysplasia index at day 50 (experiments were repeated twice with *n* = 3 microscopic fields containing viable organoids). (**E**) Dissociated cells in Matrigel (500,000 cells/100 μL) were injected s.c. into NOD-SCID mice. In vivo tumor formation 45 days later (for those without tumor formation, observation was extended for up to 3 months, experiments were repeated twice with *n* = 3). (**F**) Tumor size 45 days after s.c. implantation (*n* = 3). (**G**) H&E staining and immunofluorescence of CK20 and SMA for tumor sections. Scale bar, 100 μm. *, *p* < 0.05; ***, *p* < 0.0001 as determined with one-way ANOVA.

We further characterized the transformation effect of DMBA or/and adenovirus carrying recombinase (Ad-Cre-GFP)-mediated-PP2A deficiency in *Ppp2r1a^flox/flox^* mice-derived organoids. Similarly, DMBA had no significant effects, Ad-Cre-GFP mediated-PP2A deficiency had mild effects, and the combination of DMBA and Ad-Cre-GFP mediated-PP2A deficiency had large effects on early organoid forming efficiency, late organoid morphology, histological changes, and dysplasia (Figure 2A–D). Furthermore, only the combination of DMBA and Ad-Cre-GFP-mediated-PP2A deficiency fully endowed organoids with robust in vivo tumorigenicity (Figure 2E–G).

### 3.3. Combination of DMBA Treatment and PP2A Deficiency in Lgr5^+^ Rather than in Differentiated Villus Cells Induces Dysplasia and Oncogenic Transformation in Organoid Culture 

**Figure 3:**
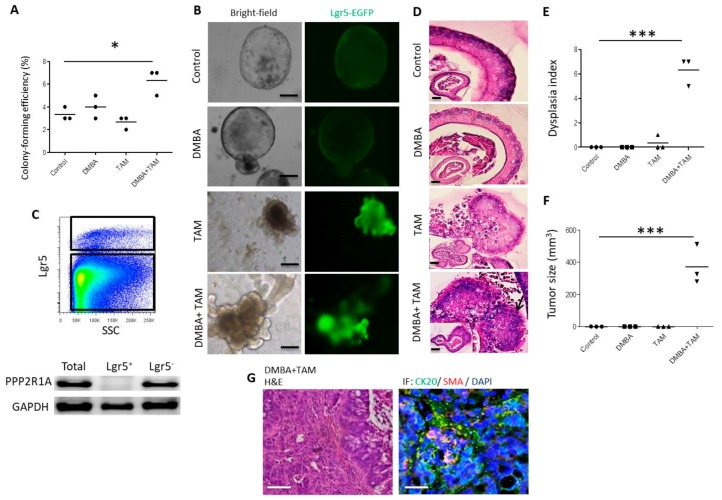
Combination of DMBA and TAM induces dysplasia and oncogenic transformation in *Lgr5-EGFP-CreERT2*; *Ppp2r1a^flox/flox^* intestinal organoid culture. In vitro culture of *Lgr5-EGFP-CreERT2; Ppp2r1a^flox/flox^* intestinal organoids without (control) or with DMBA or/and tamoxifen (TAM) in the presence of EGF, Noggin and R-spondin 1 (500 single cells/well). (**A**) Colony (organoid)-forming efficiency was calculated at day 7. At least 100 organoids were counted in each group. (**B**) Bright-field and fluorescence images of organoid culture at day 50. Scale bar, 100 μm. (**C**) Fluorescence-activated cell sorting (FACS) isolation of Lgr5^+^ and Lgr5^−^ populations. After FACS, PPP2R1A protein levels were detected by western blot. (**D**) H&E staining and histologic characterization of cystic stratified epithelium with nuclear pleomorphism (arrow). Scale bar, 50 μm. (**E**) Dysplasia index at day 50 (experiments were repeated twice with *n* = 3 microscopic fields containing viable organoids). (**F**) Dissociated cells in Matrigel (500,000 cells/100 μL) were injected s.c. into NOD-SCID mice. In vivo tumor formed 45 days later (for those without tumor formation, observation was extended for up to 3 months, experiments were repeated twice with *n* = 3). (**G**) H&E staining and immunofluorescence of CK20 and SMA for tumor sections. Scale bar, 100 μm. *, *p* < 0.05; ***, *p* < 0.0001 as determined with one-way ANOVA.

To investigate whether Lgr5^+^ crypt stem cells or differentiated villus cells serve as the cell of origin of tumors, we treated organoids from *Lgr5-EGFP-CreERT2*; *Ppp2r1a^flox/flox^* mice with DMBA or/and tamoxifen. Similarly, DMBA did not have significant effects, tamoxifen mediated-PP2A deficiency had mild effects, and the combination of DMBA and tamoxifen-mediated-PP2A deficiency had great effects on early organoid forming efficiency, late organoid morphology, histological changes, and dysplasia (Figure 3A–E). Furthermore, only the combination of DMBA and tamoxifen-mediated-PP2A deficiency endowed organoids with in vivo tumorigenicity (Figure 3F,G). Interestingly, the combination of DMBA and PP2A deficiency in organoids derived from *Villin-Cre; Ppp2r1a^flox/flox^* mice did not affect early organoid forming efficiency, late organoid morphology, histological changes, and dysplasia (Figure S2A–E), and failed to induce in vivo tumorigenicity (Figure S2F). Of note, the recombinase activity in Villin-Cre mice is gradually reduced from villus to crypt [33], nevertheless, Ppp2r1a protein is only deleted in sorted villus cells but not in sorted Lgr5^+^ cells (Figure S2A). These data suggest that Lgr5^+^ crypt stem cells but not differentiated villus cells serve as the cell of origin of intestinal tumor.

### 3.4. Combination of DMBA Treatment and PP2A Deficiency in Lgr5^+^ Cells Induces Upregulation in Stem Cell and EMT Markers, Downregulation in Differentiated Markers, and Tumorigenicity in Organoid Culture

**Figure 4:**
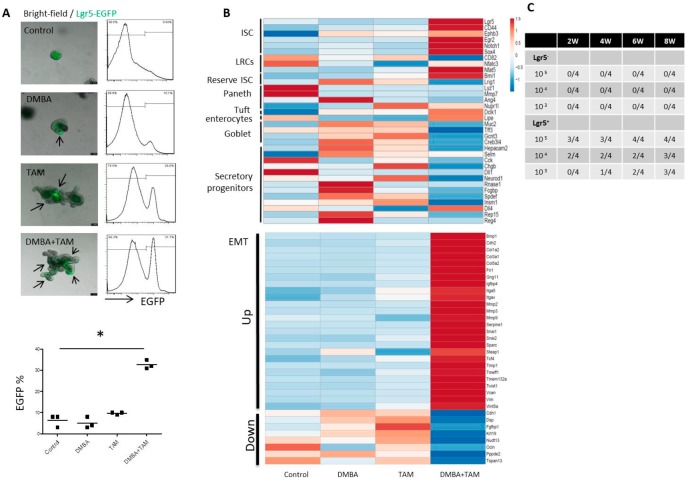
Combination of DMBA and PP2A deficient induces upregulation in stem cell and EMT markers and downregulation in differentiated markers in intestinal organoid. (**A**) In vitro culture of *Lgr5-EGFP-CreERT2; Ppp2r1a^flox/flox^* intestinal organoids. Merged bright-field and fluorescence images of organoid culture treated without (control) or with DMBA or/and tamoxifen (TAM) for 50 days in the presence of EGF, Noggin and R-spondin 1. *Lgr5*-*EGFP* were denoted as arrows. Scale bar, 100 μm. Flow cytometric analysis and quantification of GFP expression (bottom panel). No fluorescence organoid culture was serve as negative control to decide the threshold. *, *p* < 0.05 as determined with one-way ANOVA. (**B**) RNA-*sequencing* (seq) analysis of transcriptomes for 50-day organoids. The upper heat map shows clustering to previously reported RNA-seq data of sorted ISC (Intestinal Stem Cell); reserve ISC; paneth cell; LRCs (label retaining cells); tuft; enterocytes; goblet and secretory progenitor cells. The lower heat map shows clustering to EMT (epithelial–mesenchymal transition). (**C**) Tumor incidence in limiting dilution assay. Tumorigenic potential characterization of indicated numbers of Lgr5^+^ and Lgr5^−^ cells from the 50-day organoid culture. Dissociated cells in Matrigel (100 μL) were injected s.c. into NOD-SCID mice. Incidence of tumor formation was calculated more than 2 months.

Flow cytometric analysis (Figure 4A) revealed that DMBA did not increase the Lgr5^+^ cell ratio (as assayed by Lgr5-EGFP). PP2A deficiency induced a marginal increase, while the combination of DMBA and PP2A deficiency induced a significant increase in the Lgr5^+^ cell ratio. Comparative gene expression analysis of RNA samples isolated from organoid culture of *Lgr5-EGFP-CreERT2; Ppp2r1a^flox/flox^* mice 50 days after DMBA and tamoxifen administration revealed marked upregulation of stem cell genes, such as *Lgr5*, *CD44*, *Ephb3*, *Egr2*, *Notch1*, and *Sox4*; as well as EMT markers, such as *Snail1*, *Snail2*, *Twist1*, *fibronectin*, and *vimentin*; and a marked downregulation of genes associated with differentiated cells, such as Paneth, enterocyte, goblet, and secretory cells compared to other treatment groups (Figure 4B). Furthermore, a small number (10^3^) of Lgr5^+^ but not Lgr5^−^ cells isolated from organoid culture treated with DMBA and tamoxifen possessed in vivo tumorigenicity (Figure 4C). Collectively, these data suggest that the combination of DMBA and PP2A deficiency converted Lgr5^+^ crypt stem cells into CSCs.

### 3.5. Combination of DMBA Treatment and PP2A Deficiency Generates CSCs through PI3K, ERK, and Wnt Activation

**Figure 5:**
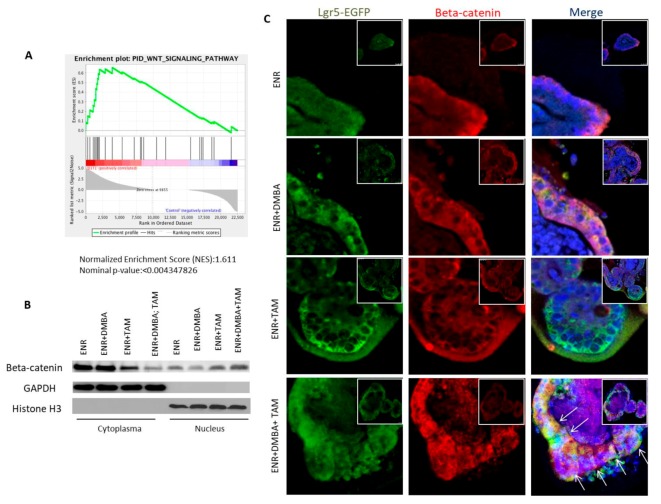

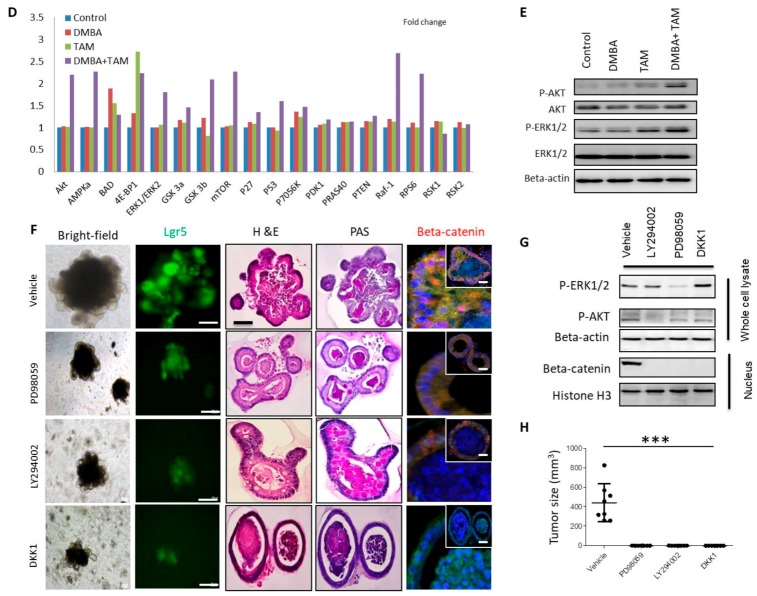
Beta-catenin activation caused by PI3K and ERK mediates dysplasia and oncogenic transformation in organoid culture. *Lgr5-EGFP-CreERT2; Ppp2r1a^flox/flox^* intestinal organoids were treated without (control) or with DMBA or/and tamoxifen (TAM) for 50 days in the presence of EGF, Noggin and R-spondin 1. (**A**) Gene set enrichment analysis (GSEA) shows “WNT SIGNALING PATHWAY” for the organoids treated with DMBA and TAM group versus control group, *p* < 0.05. (**B**) Western blotting of nuclear and cytoplasmic fractions. GAPDH and Histone H3 were used as protein loading controls for the cytoplasmic and nuclear fractions, respectively. (**C**) Immunofluorescence of Lgr5-EGFP and beta-catenin expression in organoid (denoted by arrows). Scale bar, 100 μm. (**D**) Graphs of mouse AKT pathway phosphorylation protein expression array (original data in Extended Data Figure 3) and densitometric analyses, *n* = 1. (**E**) Western blotting. (**F**) Bright-field, fluorescence images, H&E, PAS staining and beta-catenin immunofluorescence images. (**G**) Western blot analysis of whole cell lysate and nuclear fraction from organoid cultures treated with EGF, Noggin, R-spondin 1 (ENR), DMBA, and TAM for 50 days in the absence (Vehicle) or presence of indicated inhibitor treatment. (**H**) Dissociated cells in Matrigel (500,000 cells/100 μL) were injected s.c. into NOD-SCID mice. In vivo tumor formed 45 days later (For those without tumor formation, observation was extended for up to 3 months, *n* = 8). ***, *p* < 0.0001 as determined with one-way ANOVA.

To understand the molecular characteristics of the genes and pathways involved in organoid culture of *Lgr5-EGFP-CreERT2*; *Ppp2r1a^flox/flox^* mice after DMBA and tamoxifen administration, RNA-seq and gene set enrichment analysis (GSEA) were performed. Similar to previous findings that intestinal tumor begins with specific molecular alterations in Wnt-beta-catenin pathway [13,23], Wnt signaling was upregulated upon oncogenic transformation of organoid culture (Figure 5A). Western blotting of nuclear proteins (Figure 5B) and immunofluorescence (Figure 5C) revealed that nuclear accumulation of beta-catenin was predominantly observed in organoids with both of DMBA treatment and PP2A deficiency. Screening with a serine/threonine phosphorylation protein array (Figure S3) followed by confirmation with western blotting further revealed that PI3K/AKT/GSK-3beta and Raf/ERK were activated in organoids with both DMBA treatment and PP2A depletion compared to other treatment groups (Figure 5D,E). Interestingly, treatment with the PI3K inhibitor LY294002, MEK inhibitor PD98059, and Wnt inhibitor DKK1 reduced the formation of malformed organoids, inhibited dysplasia (Figure 5F,G), and completely blocked in vivo tumorigenicity (Figure 5H). Notably, not only the WNT signaling was the most, but also the PI3K and RAS-MAPK (ERK) signalings were common altered pathways in human intestinal tumor, as revealed by The Cancer Genome Atlas (TCGA) Project [14]. These data suggest that CSC generation by DMBA treatment and PP2A deficiency depends on the activation of PI3K, ERK, and Wnt signals. 

### 3.6. Lgr5^+^ CSCs Are R-Spondin 1-Dependent

**Figure 6:**
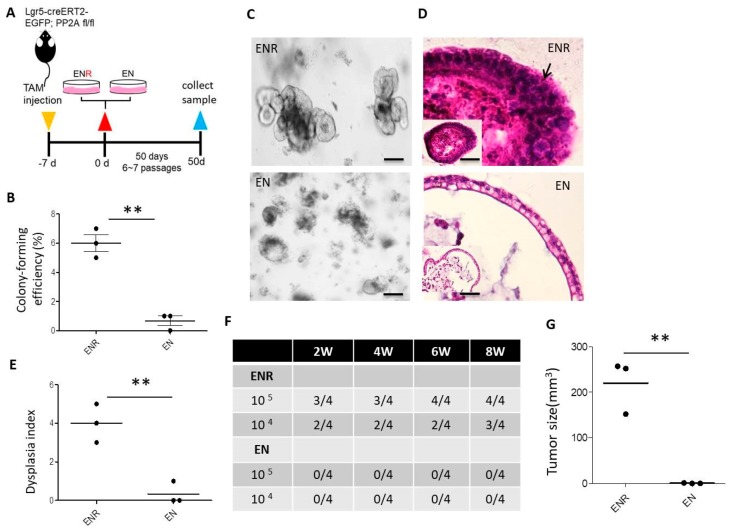
R-spondin 1 drives Wnt-dependent dysplasia and oncogenic transformation in the intestinal organoid culture. In vitro culture of *Lgr5-EGFP-CreERT2; Ppp2r1a^flox/flox^* intestinal organoids (500 single cells/well) treated with DMBA and tamoxifen (TAM) in the presence of EGF, Noggin, and with (ENR) or without R-spondin-1 (EN). (**A**) Schematic illustration of the experimental design. (**B**) Colony (organoid)-forming efficiency at day 7. At least 100 organoids were counted in each group. (**C**) Bright-field images at day 50. Scale bar, 100 μm. (**D**) H&E staining and histologic characterization of cystic stratified epithelium with nuclear pleomorphism (arrow). Scale bar, 50 μm. (**E**) Dysplasia index at day 50 (experiment was repeated twice with *n* = 3 microscopic fields containing viable organoids). (**F**) Incidence of tumor formation at indicated time periods, and (**G**) tumor size in NOD-SCID mice after s.c. injection 45 days later of indicated cell numbers from 50-day organoid. **, *p* < 0.01 as determined with Student’s *t*-test.

CSCs isolated from human intestinal tumor specimens express the Lgr5 crypt marker [34]. R-spondin is expressed by the intestinal stroma and is differentially upregulated during *Citrobacter rodentium*- and dextran sulfate sodium (DSS)-induced colitis in mice, which reflects human ulcerative colitis, a precancerous stage [35]. These data suggest a role for R-spondin 1 and its receptor Lgr5 in the maintenance of undifferentiated status and tumorigenesis of human colorectal CSCs. In contrast to tumorigenesis initiated by dedifferentiation (tumor generated from intestinal epithelial cell of *villin-creER^T2^/APC ^lox/lox^/K-ras^G12D/+^* mice) [4], where tumor cells were Lgr5^−^ and generated independently of R-spondin 1, tumor cells in the current study were Lgr5^+^ and generated dependently on R-spondin 1 (Figure 6), suggesting that tumor cells generated by DMBA treatment and PP2A deficiency in mouse Lgr5^+^ cells were indeed intestinal CSCs and could serve as surrogates of human colorectal CSCs [34]. Previous reports using tumorigenesis models generated by genetic manipulation in differentiated villus cells, even with low efficiency in tumorigenesis [27], also demonstrated important roles for Lgr5^+^ cells in tumorigenesis/metastasis [7,8].

### 3.7. Combination of DMBA Treatment with PP2A Deficiency in Lgr5^+^ Also Induces Tumor Formation In Vivo

**Figure 7:**
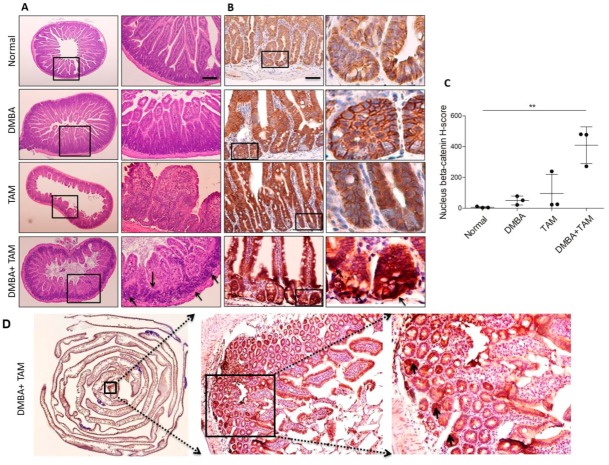
Combination of DMBA treatment with PP2A deficient in Lgr5^+^ drives intestinal neoplasia in both the small intestine and colon. (**A**) H&E staining and (**B**) Beta-catenin IHC were performed on the serial sections. Multiple beta-catenin^high^ adenomas were observed throughout the colon 36 days after induction. (**C**) Quantitative analysis of nucleus beta-catenin H-score from (**B**). (**D**) High level expression of beta-catenin was apparent in the transformed stem cell compartment (Peyer’s patches are stained blue). Multiple beta-catenin^high^ transformed cells were observed throughout the intestinal 36 day after induction (magnified at right panel, denoted by arrows). Original magnifications: (**A**) left, 4×; (**A**) right, 10×; (**B**) left, 20×; (**B**) right, 40×; (**D**) center, 20×; (**D**) right, 40×; Scale bar, 100 μm. **, *p* < 0.01 as determined with one-way ANOVA.

We investigated whether tumorigenesis generated by DMBA treatment and PP2A deficiency in mouse Lgr5^+^ cells reflects tumor formation in vivo. *Lgr5-EGFP-CreERT2; Ppp2r1a^flox/flox^* mice treated with DMBA and tamoxifen formed adenocarcinoma in the small intestine and colorectal regions 36 days later. Histological analysis revealed that mice receiving DMBA and tamoxifen increased the incidence of multiple foci adenoma compared to other groups (Figure 7A). IHC analysis revealed that beta-catenin accumulated in the nuclei of tumor cells mainly in the crypt area (Figure 7B) and quantitative evaluation of nucleus beta-catenin accumulation [29] further showed that nucleus beta-catenin H-score was significantly greater in mice receiving DMBA and tamoxifen compared to other groups (Figure 7C,D). The efficiency and rapidity were much greater than tumorigenesis generated by *Apc* deficiency in either Lgr5^+^ crypt stem cell- [3] or differentiated villus cell-based models [4].

### 3.8. Not Only DMBA but Also Other Carcinogens Induce Tumors from Lgr5^+^ Intestinal Stem Cells of PP2A Deficient Mice

To demonstrate carcinogen-induced tumor from Lgr5^+^ intestinal stem cells of PP2A deficient mice was not limited to DMBA, *N*-methyl-*N*-nitrosourea (MNU) and 2-amino-1-methyl-6-phenylimidazo-[4,5-b]pyridine (PhIP) were also administered in combination with tamoxifen treatment in organoid cultures derived from *Lgr5-EGFP-CreERT2; Ppp2r1a^flox/flox^* mice and from *Villin-Cre; Ppp2r1a^flox/flox^* mice. Interestingly, increased early organoid forming efficiency, late organoid with irregular nuclei and prominent nucleoli morphology and dysplasia, and in vivo tumorigenicity were only observed in organoid cultures derived from *Lgr5-EGFP-CreERT2; Ppp2r1a^flox/flox^* mice but not from *Villin-Cre; Ppp2r1a^flox/flox^* mice (Figure S4A,B). More importantly, we found that tumorigenicity induced by combination of MUN or PhIP with PP2A deficiency also depended on the activation of PI3K, ERK, and Wnt signals (Figure S4C–F). Similarly, combination of MUN or PhIP treatment with PP2A deficiency in Lgr5^+^ cells also induced tumor formation in vivo (Figure S4G).

### 3.9. Mutational Landscapes of Intestinal Tumors Derived from Lgr5^+^ Cells Treated with Carcinogen and PP2A Deficiency

**Figure 8:**
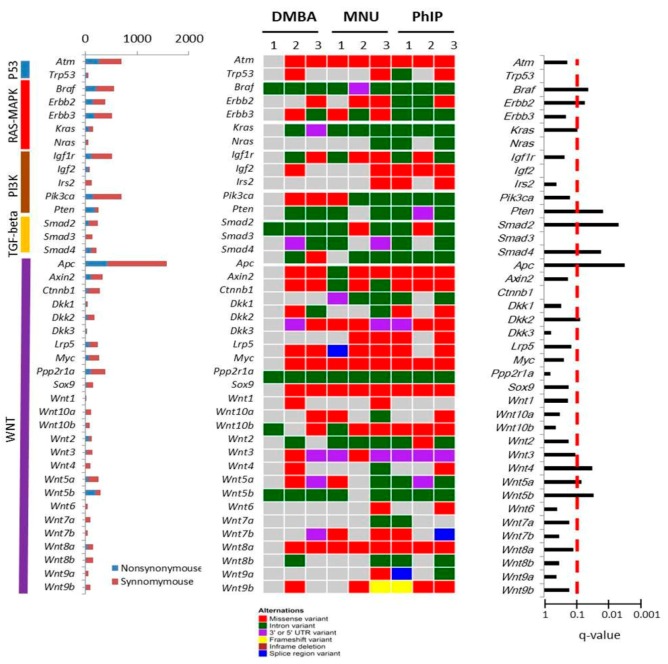
Landscape of somatic mutant genes in three carcinogen treated organoid cultures. Data matrix shows number of somatic mutant genes in each carcinogen treated organoid cultures and were classified according to their pathway. Somatic mutations are presented according to the type of mutation (missense variant, intron variant, 3′ or 5′ prime UTR variant, frameshift variant, inframe deletion, or splice region variant) On the left, the total number of mutations of each gene within all three groups is shown with a bar plot, while the q-value of each significantly mutated gene is shown on the right.

To gain insight of the genetic alterations that drove these pathways in the models, we performed whole-exome sequencing (WES) (Figure S5A). Somatic variants in each chemical induced tumor organoid sample were identified with a tumor-control paired strategy by removing the variants in their paired control samples and the variants affecting protein coding sequence were further filtrated (Figure S5B). The numbers of somatic mutations, including synonymous and nonsynonymous mutations, for organoid cultures with combined DMBA, MUN, or PhIP treatment with PP2A deficiency in Lgr5^+^ cells were shown in Figure S5C. IPA analysis of 270 mutated genes shared by DMBA, MUN, and PhIP treatment with PP2A deficiency in Lgr5^+^ cells (Figure S5D) revealed 10 top significantly enriched pathways (Figure S5E), including the intestinal tumor metastasis signaling (*p*-value = 2.12 × 10^−57^) and Wnt/beta-catenin signaling (*p*-value = 1.40 × 10^−55^). Furthermore, beta-catenin (*CTNNB1*) seemed to be a common downstream hub as identified by the Path Explorer function in IPA (Figure S5F). There were five core pathways related with human intestinal tumor found by TCGA, including p53, RAS-MAPK, PI3K, TGF-beta, and WNT pathways (Figure 8) [14]. We identified several recurrent mutations with FDR < 0.1, including *Braf*, *erbb2*, *kras*, *pten*, *Smad2*, *Smad4*, *Apc*, *DKK2*, *Wnt4*, *Wnt5a*, and *Wnt5b* (Figure 8). Based on these analyses, the mouse intestinal tumor organoid models based on combined use of chemical carcinogen and genetic modification can recapitulate the human colorectal cancer development process in response to complex interactions between the genotype and environmental factor.

## 4. Discussion

To improve the efficiency of tumorigenesis in GEMMs, multiple gene mutations are necessary. Given that the frequency of point mutations varies from less than 0.1 to greater than 50 mutations per megabase [36], GEMMs are a greatly oversimplified view of the numbers and types of mutations found in human cancers [37]. The use of GEMMs in combination with carcinogenesis increased the tumor spectrum or speeded the tumor formation observed in some GEMMs, such as the *p53*^−/−^ mouse model in combination with exposure to carcinogens or radiation [21,38]. In the current study, P*pp2r1a^−/−^* mouse model in combination with exposure to different carcinogens induced tumorigenesis through similar signaling pathways, while GEMM or carcinogen exposure alone did not induce tumorigenesis. These data suggest that the combination of GEMMs with exposure to carcinogen or other environmental agents is a logical approach in studying tumorigenesis and cancer progression.

PP2A is a tumor suppressor that regulates many oncogenic pathways. In fact, decreased PP2A activity has been reported as a common event in colorectal cancer [39]. DMBA and MNU are important environmental carcinogens. PhIP, a food-borne carcinogen produced while cooking meat and fish, models human colon cancer in rodents [40]. Here, we demonstrated that DMBA, MNU, or PhIP each induced intestinal organoid transformation when combined with PP2A deficiency in Lgr5^+^ intestinal stem cells but not in differentiated villus cells, suggesting that PP2A plays a role in suppressing colorectal tumorigenesis induced by chemical carcinogen exposure. Our results provide further experimental evidence to demonstrate that the cell of origin of intestinal tumor is crypt stem cells instead of differentiated villus cells. We did not focus on the molecular mechanism underlying the differential tumorigenicity pathways between stem cells and differentiated cells, which requires further investigation.

A recent study shows that unless the R-spondin and Wnt ligands are both present, the default fate of Lgr5^+^ crypt stem cells is differentiation [41]. However, gain-of-function studies reveal that R-spondin and Wnt ligands have qualitatively distinct, non-interchangeable roles in crypt stem cells. Wnt proteins confer a basal competency unto the Lgr5^+^ crypt stem cell by inducing R-spondin receptor expression that enables R-spondin-driven Lgr5^+^ crypt stem cell self-renewal. In the current study, using a combination of carcinogen treatment and PP2A deficiency, intestinal organoids derived from Lgr5-cre; Ppp2r1aflox/flox, but not Villin-Cre; Ppp2r1aflox/flox, underwent oncogenic transformation and exhibited CSC phenotypes that were dependent on the presence of R-spondin 1 in the culture media. These studies together suggest the important roles of R-spondin signaling in stem cell self-renewal and preventing differentiation. The discrepancy of R-spondin 1 dependence between tumorigenicity induced by the current method and previous GEMM relied on *Apc* depletion or *CTNNB1* activation [4] is supported by the mutual exclusion of *R-spondin* fusion and *Apc* or *CTNNB1* mutation identified in human intestinal tumors [20]. The Lgr5/R-spondin 1 complex degrades Rnf43 and Znrf3, two transmembrane E3 ligases that remove Wnt receptors from the stem cell surface. Consistently, simultaneous deficient of these two E3 ligases in the intestinal epithelium induced the formation of unusual adenomas consisting entirely of Lgr5^+^ stem cells and their niche [42].

Simultaneous carcinogen treatment and conditional deletion of PP2A in villus cells did not induce transformation or increased proliferation or dysplasia of intestine organoids. The current study did not aim to study the underlying mechanism that differentiates Lgr5^+^ crypt stem cells from differentiated villus cells in terms of vulnerability to carcinogenesis. Based on our current results, both Wnt and Rspondin/Lgr5 signaling pathways play important roles in nuclear-beta catenin localization, which is important for stem cell self-renewal and may also initiate tumorigenesis once dysregulated.

## 5. Conclusions

In summary, we demonstrated that carcinogen-induced cancer arises from Lgr5^+^ crypt stem cells in *Ppp2r1a*^−/−^ mice. In addition, combing carcinogenesis with GEMM recapitulated the developmental process of environmentally induced human tumor, while increasing the rate and percentage of tumorigenesis. Interestingly, exposure to different carcinogens, such as DMBA, MNU, or PhIP, when combined with PP2A deficiency in Lgr5^+^ intestinal stem cells induced tumorigenesis that was dependent on the activation of pathways including Wnt, PI3K, and RAS-MAPK signalings, the common altered pathways revealed by TCGA human intestinal tumor project [14]. Together, these data suggest that PP2A functions as a tumor suppressor in intestine carcinogenesis. This organoid platform provides experimental evidence as to its usefulness in detection of key oncogenes and suppressor genes as early molecular epidemiological biomarkers of carcinogenesis, and is useful in human cancer prevention practice as well. 

## Supplementary Materials

The following are available online at https://www.mdpi.com/2073-4409/9/1/90/s1, Figure S1: Screening strategy for phosphatase hub gene in TCGA-COAD data set, Figure S2: DMBA and PP2A deficiency did not induce dysplasia and oncogenic transformation in *Villin-Cre; Ppp2r1a^flox/flox^* intestinal organoid culture, Figure S3: Serine/threonine phosphorylation protein array screening in individual organoid groups, Figure S4: Combination of MNU or PhIP and TAM induces dysplasia and oncogenic transformation in *Lgr5-EGFP-CreERT2; Ppp2r1a^flox/flox^* intestinal organoid culture, Figure S5: Ingenuity pathway analysis (IPA) to identify significant canonical pathways and interactome in three carcinogens induced oncogenic transformation in *Lgr5-EGFP-CreERT2; Ppp2r1a^flox/flox^* intestinal organoids.
