# Supplementary material for: PP2A Deficiency Enhances Carcinogenesis of Lgr5+ Intestinal Stem Cells Both in Organoids and In Vivo
Source: Cells. 2019 Dec 30;9(1):90. doi: 10.3390/cells9010090 (PMC7017129; doi:10.3390/cells9010090)
Supplement: Supplementary file 1 [file cells-09-00090-s001.zip › cells-657216-SI.pdf]

PP2A Deficiency Enhances Carcinogenesis of Lgr5^{+} Intestinal Stem Cells both in Organoids and In Vivo

Supplementary Figures

a

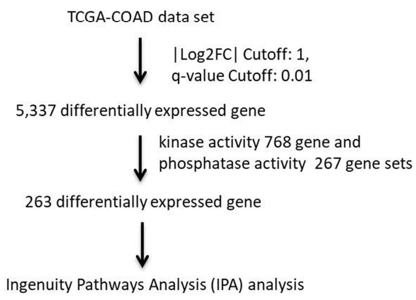

c

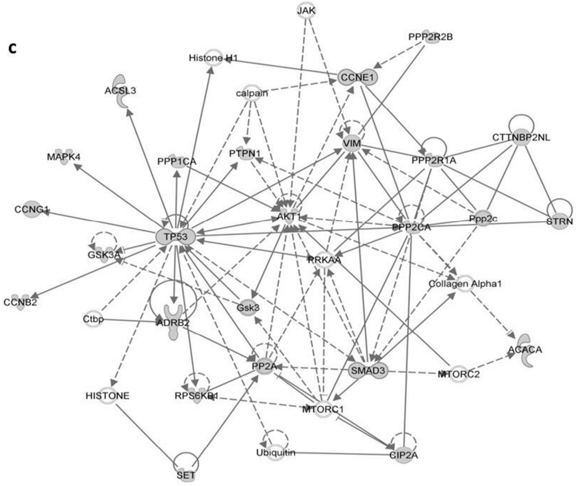

b

Top Diseases and Functions	Score
1. Post-Translational Modification, Cell Death and Survival, Cellular Function and Maintenance	43
2. Cell Cycle, Post-Translational Modification, Nutritional Disease	36
3. Cancer, Organismal Injury and Abnormalities, Tumor Morphology	34
4. Post-Translational Modification, Amino Acid Metabolism, Small Molecule Biochemistry	30
5. Cancer, Hematological Disease, Immunological Disease	24

d

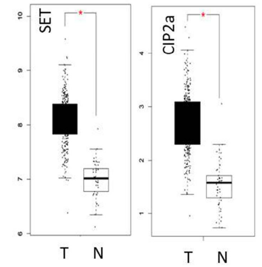

Figure S1. Screening strategy for phosphatase hub gene in TCGA-COAD data set. (**A**) Flow chart depicting the strategy used to identify hub genes for IPA analyses. (**B**) Top ranked networks identified by IPA. IPA networks that depict associations between genes involved in certain biological functions: (**1**) Post-Translational Modification, Cell Death and Survival, Cellular Function and Maintenance (score 43), (**2**) Cell Cycle, Post-Translational Modification, Nutritional Disease (score 36), (**3**) Cancer, Organismal Injury and Abnormalities, Tumor Morphology (score 34), (**4**) Post-Translational Modification, Amino Acid Metabolism, Small Molecule Biochemistry (score 30). (**5**) Cancer, Hematological Disease, Immunological Disease (score 24). (**C**) The network is graphically displayed with genes/gene products as nodes and the biological relationships between the nodes as edges (lines). The diagrams show direct (solid lines) and indirect (dashed lines) interactions between genes known to orchestrate common functions. The length of an edge reflects the evidence in the literature supporting that node-to-node relationship. The score is derived from a p value and indicates the likelihood of the focus genes in a network to be found together due to random chance. (**D**) Analyses of RNA-seq data to TCGA of colorectal cancer reveal increase of SET or CIP2A in tumors from the colorectal cancer samples in comparison with their corresponding non-tumor parts.

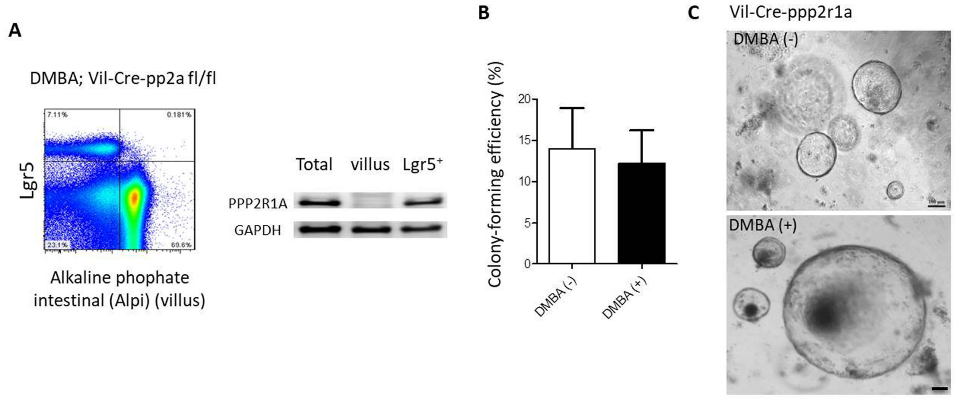

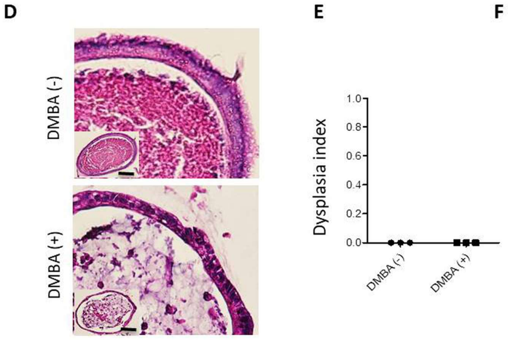

F

	2W	4W	6W	8W
**Vil-cre-pp2a fl/fl**				
10 5	0/4	0/4	0/4	0/4
10 4	0/4	0/4	0/4	0/4
10 3	0/4	0/4	0/4	0/4
**DMBA; Vil-cre-pp2a fl/fl**				
10 5	0/4	0/4	0/4	0/4
10 4	0/4	0/4	0/4	0/4
10 3	0/4	0/4	0/4	0/4

Figure S2. DMBA and PP2A deficiency did not induce dysplasia and oncogenic transformation in *Villin-Cre; Ppp2r1a$^{flox/flox}$* intestinal organoid culture. (**A**) FACS isolation of alkaline phosphate intestinal (Alpi)$^{+}$ve populations. Mice were induced Ppp2r1a deficiency specifically in villus cells. After FACS, PPP2R1A protein was detected by western blotting. (**B**–**F**) In vitro culture of *Villin-Cre; Ppp2r1a$^{flox/flox}$* intestinal organoids treated without or with DMBA in the presence of EGF, Noggin and R-spondin 1 (500 single cells/well). (**B**) Colony (organoid)-forming size was calculated at day 7. * $p < 0.05$ as determined with student's t-test. Percentage of actively growing organoids that exhibited at least two budding structures at day 7. At least 100 organoids were counted in each group. (**C**) Bright-field images at day 50. Scale bar, 100 μm. (**D**) H&E staining and histologic characterization of cystic epithelium. Scale bar, 50 μm. (**E**) Dysplasia index at day 50 (n = 3 microscopic fields containing viable organoids). (**F**) Dissociated cells in Matrigel (100 μL) were injected s.c. into NOD-SCID mice. Incidence of in vivo tumour formation at indicated time period (For those without tumour formation, observation was extended for up to 3 months).

A

Each antibody is spotted in duplicate vertically

Row								
1<br>2	POS	POS	NEG	NEG	Akt (P-Ser473)	AMPKa (P-Thr172)	BAD (P-Ser112)	4E-BP1 (P-Thr36)
3<br>4	ERK1 (P-T202/Y204)<br>ERK2 (P-Y185/Y187)	GSK3a (P-Ser21)	GSK3b (P-Ser9)	mTOR (P-Ser2448)	p27 (P-Thr198)	P53 (P-Ser15)	P70S6K (P-Thr421/Ser424)	PDK1 (P-Ser241)
5<br>6	PRAS40 (P-Thr246)	PTEN (P-Ser380)	Raf-1 (Ser301)	RPS6 (P-Ser235/236)	RSK1 (P-Ser380)	RSK2 (P-Ser386)	NEG	POS

POS = Positive Control Spot
NEG = Negative Control Spot

B

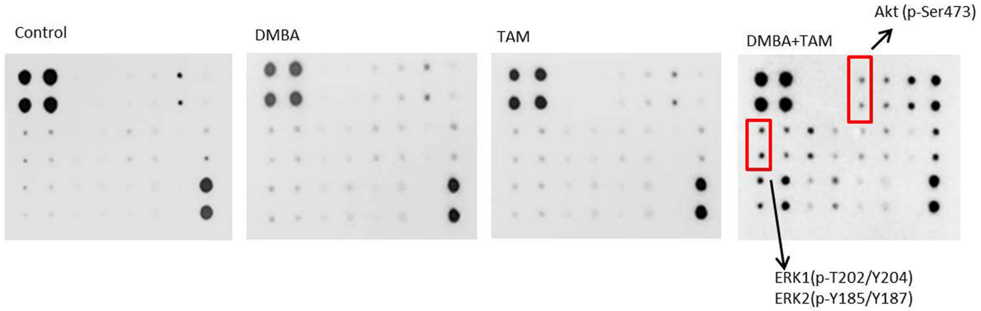

Figure S3. Serine/threonine phosphorylation protein array screening in individual organoid groups. (**A**) List and illustration of protein expression array. Phosphorylated forms of these proteins were measured by applying organoid cell lysates to the mouse AKT pathway phosphorylation array (RayBiotech), and were processed according to the manufacturer's protocol. (**B**) *Lgr5-EGFP-CreERT2; Ppp2r1a$^{flox/flox}$* intestinal organoids (500 single cells/well) treated without (Control) or with DMBA or/and tamoxifen (TAM) for 50 days in the presence of EGF, Noggin and R-spondin 1 and harvested for analysis. Blots are representative arrays in individual organoid lysates.

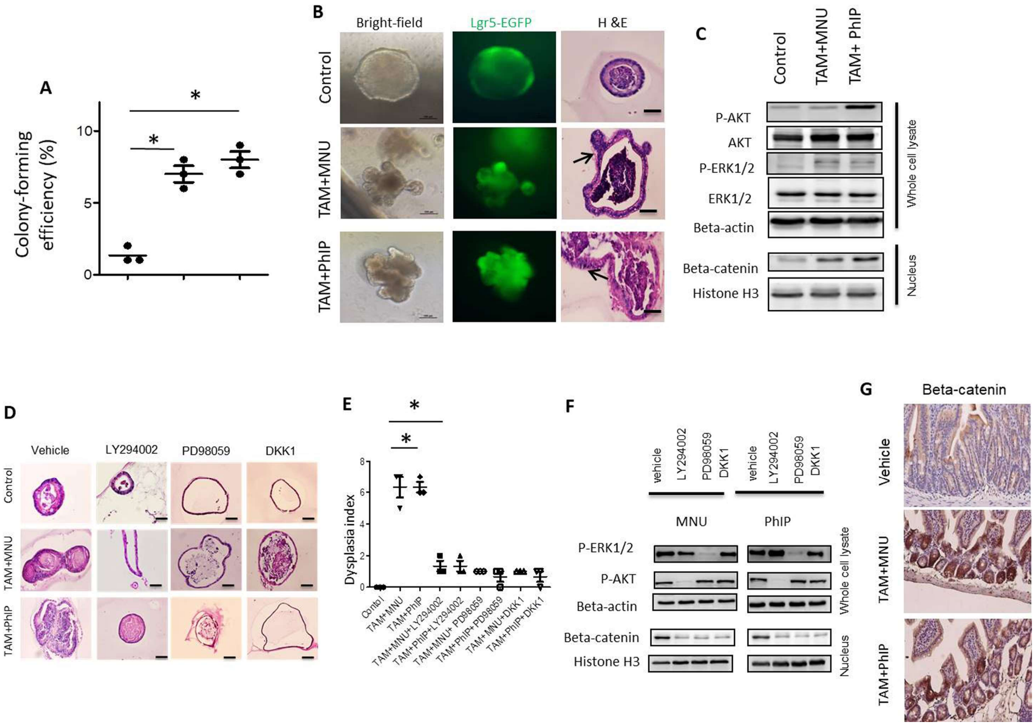

Figure S4. Combination of MNU or PhIP and TAM induces dysplasia and oncogenic transformation in *Lgr5-EGFP-CreERT2; $Ppp2r1a^{flox/flox}$* intestinal organoid culture. In vitro culture of *Lgr5-EGFP-CreERT2; $Ppp2r1a^{flox/flox}$* intestinal organoids treated without (Control) or with MNU or PhIP and tamoxifen (TAM) for 7 days in the presence of EGF, Noggin and R-spondin 1 (500 single cells/well). (**A**) Colony (organoid)-forming efficiency was calculated at day 7. Percentage of actively growing organoids that exhibited at least two budding structures. At least 100 organoids were counted in each group at day 7. (**B**) Bright-field, fluorescence images and H&E staining of organoid cultured for 50 days. Histologic characterization of cystic stratified epithelium with nuclear pleomorphism are shown (arrows). Scale bar, 100 µm. (**C**) Western blot. (**D**) H&E staining and histologic characterization of cystic epithelium. Scale bar, 100 µm. (**E**) Dysplasia index calculated at day 50 (n = 3 microscopic fields containing viable organoids). * $p < 0.05$ as determined with One-Way ANOVA. (**F**) Western blot analysis of whole cell lysates and nuclear enriched fractions of organoids treated with individual inhibitor. (**G**) Beta-catenin IHC were performed on sections in each group. Multiple beta-catenin^{high} adenomas were observed throughout the colon 36 days after induction.

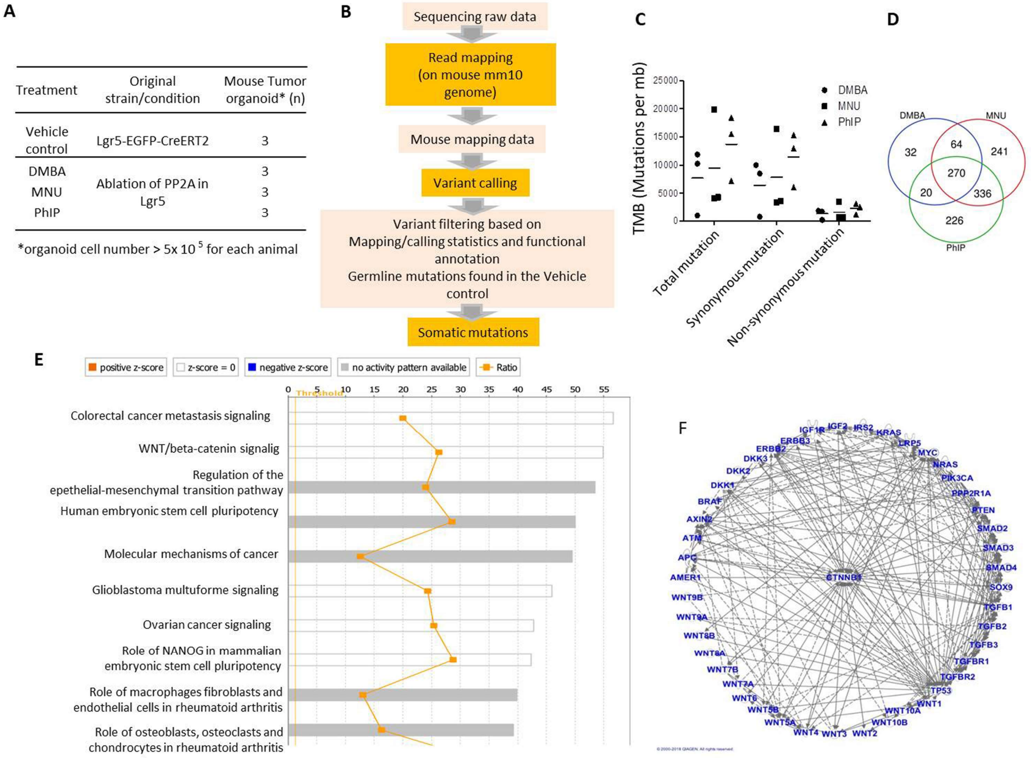

Figure S5. Ingenuity pathway analysis (IPA) to identify significant canonical pathways and interactome in three carcinogens induced oncogenic transformation in *Lgr5-EGFP-CreERT2; Ppp2r1a$^{flox/flox}$* intestinal organoids. (**A**) The organoid cultures are expanded for 50 day to obtain sufficient DNA for whole-exome sequencing analysis. A panel of control samples was also subjected to whole-exome sequencing to exclude germline variants, "n" represent different animals. (**B**) Bioinformatics analysis pipeline for somatic mutation detection. The most frequently mutated gene in The Cancer Genome Atlas Colon Adenocarcinoma (TCGA-COAD) data sets were used as a reference in this analysis. (**C**) The numbers of gene mutation in three carcinogen treated organoid cultures. (**D**) Venn diagram of nonsynonymous mutation among three carcinogens treated organoid cultures. (**E**) The 270 nonsynonymous mutations found in all three carcinogens treated organoid cultures were subjected to IPA analysis for identification of the top 10 significantly enriched pathways. Pathways identified are represented on the y-axis. The x-axis corresponds to the –log of the *p*-value (Fisher's exact test) and the x-axis represents the ratio (orange points) of the number of genes. (**F**) Interactomic analysis by IPA software. The network is displayed graphically as nodes (CTNNB1) and edges (the biological relationships between nodes).
